# Carbide clusterfullerene DyYTiC@C_80_ featuring three different metals in the endohedral cluster and its single-ion magnetism†

**DOI:** 10.1039/c8cc04736g

**Published:** 2018-09-20

**Authors:** Ariane Brandenburg, Denis S. Krylov, Alexander Beger, Anja U. B. Wolter, Bernd Büchner, Alexey A. Popov

**Affiliations:** Leibniz Institute for Solid State and Materials Research (IFW), D-01069 Dresden, Germany

## Abstract

Carbide clusterfullerene DyYTiC@C_80_-*I*_h_ with three different metal atoms in the endohedral cluster is obtained by arc-discharge synthesis with methane as reactive gas and is successfully isolated by HPLC. The compound shows single-molecule magnetism (SMM) with magnetic hysteresis below 8 K. The SMM properties of DyYTiC@C_80_ are compared to those of DySc_2_N@C_80_ and the influence of the central atom in the endohedral cluster is analyzed.

A possibility of combining different types of metal atoms within one endohedral metallofullerene (EMF) molecule gives new insight into the chemical and physical properties of EMFs.^1^ For instance, combination of metals with different ionic radii can substantially change the yield or structural, chemical, or electrochemical properties of EMFs.^2^ A combination of metals with different contrast properties can be beneficial for biomedical applications,^3^ whereas “substitution” of lanthanides with diamagnetic metals has a dramatic influence on the magnetic properties of EMFs.^4^

The vast majority of mixed-metal EMFs are nitride clusterfullerenes, M_3_N@C_2*n*_, where M is usually a trivalent metal such as Sc, Y, or lanthanides. Sc-based mixed-metal nitride clusterfullerenes have been especially popular, and Sc–Y,^2*n*^ Sc–Ti,^5^ Sc–V,^2*l*^ and numerous Sc–lanthanide binary systems have been synthesized.^2*a*,*d*,*e*,*g*,*i*,*j*,6^ The list of studied non-Sc mixed-metal nitride clusterfullerenes is also rather long and includes Ti–Y,^7^ Ce–Y,^2*d*,*e*^ Ce–Lu,^2*h*,8^ Gd–Ho,^3*b*^ Gd–Lu,^3^ Ho–Y,^4*c*^ Ho–Lu,^3*a*,4*c*^ and Lu–Y^9^ binary systems. Mixed-metal EMFs with the same fullerene cage but different cluster compositions may have very similar retention times, which complicates their chromatographic separation. For ternary systems the complexity is increasing dramatically, and only one EMF with a ternary-metal cluster, ScYErN@C_80_, has been reported so far.^10^

M_2_TiC@C_2*n*_ is a special type of trimetal carbide clusterfullerene,^2*f*,11^ which exists only as a mixed-metal EMF. The composition of the carbide cluster is fixed by Ti, which forms a double bond with the central carbon atom; M is thus a trivalent metal such as Sc, Y, or a lanthanide.^2*f*,11^ The variation of the cluster composition is not possible, *i.e.* formation of carbide clusterfullerenes with Ti_3_C, MTi_2_C, or M_3_C clusters has not been detected, which dramatically simplifies the isolation. If methane is used as a reactive gas in the arc-discharge synthesis, lanthanide-based M_2_TiC@C_80_-*I*_h_ can be obtained with a high degree of selectivity.^11*a*^ The carbide cluster in M_2_TiC@C_80_ is isoelectronic and isostructural to the nitride cluster in M_2_ScN@C_80_, and both types of clusters have very similar charge distribution.^11*b*^ Similar to Dy_2_ScN@C_80_,^12^ Dy_2_TiC@C_80_ has been found to be a single molecule magnet (SMM),^11*a*^ but considerably softer than the former. In this work we show that this Ti-carbide template enables facile access to mixed-metal EMFs with three different metals in the cluster, synthesize DyYTiC@C_80_ and analyse its magnetic properties in comparison to DySc_2_N@C_80_, which was studied in detail earlier.^13^

EMF-containing soots were obtained by arc-discharge syntheses with graphite electrodes filled with the mixture of Dy, Y, Ti, and graphite powder (molar ratio 0.5 : 0.5 : 1: 12.5). The atmosphere in the reactor contained a mixture of He (237 mbar) and CH_4_ (13 mbar).^2*f*,11*a*^ After pre-extraction with acetone, the soot was Soxhlet-extracted with CS_2_ for 20 hours. A typical chromatogram of the fullerene extract obtained in these synthesis conditions is shown in Fig. 1. Based on mass-spectrometric analysis (laser-desorption ionization, LDI), the compounds with retention times less than 30 min are assigned to empty fullerenes (C_60_, C_70_, C_84_ *etc.*). In the range of endohedral metallofullerenes (*t* > 30 min), two main chromatographic peaks are observed, denoted as **F1** and **F2** (Fig. 1). The dominant EMF fraction **F1** (36–39 min) is found to be a mixture of three M_2_TiC@C_80_ EMFs (M_2_ = Dy_2_, Y_2_, and DyY). Mass-spectral analysis of the fraction **F2** (39–42 min) shows the presence of M_2_TiC@C_80_ and M_2_TiC_2_@C_80_ (M_2_ = Dy_2_, Y_2_, and DyY).

Based on the previous studies of the synthesis of lanthanidetitan carbide clusterfullerenes,^2*f*,11*a*^ the fullerenes in fraction **F1** can be identified as M_2_TiC@C_80_ with the *I*_h_-symmetric cage isomer, whereas those in fraction **F2** are identified as *D*_5h_ cage isomers of M_2_TiC@C_80_ and *I*_h_ isomers of M_2_TiC_2_@C_80_.

Fraction **F1** was further subjected to recycling HPLC, which afforded the separation of Y_2_TiC@C_80_-*I*_h_, DyYTiC@C_80_-*I*_h_, and Dy_2_TiC@C_80_-*I*_h_ after 11 cycles (Fig. 2a). UV-vis-NIR absorption spectra of all three EMFs are virtually identical (Fig. 2b), which proves that they are isostructural. The spectra exhibit the characteristic absorption pattern observed earlier for M_2_TiC@C_80_ with the *I*_h_ cage isomer (the metal M has no significant influence on the spectra).^11*a*,*b*^ Hence the molecular structure of the newly isolated DyYTiC@C_80_ can be unequivocally assigned to the C_80_-*I*_h_ cage (Fig. 2c).

The relative yield of M_2_TiC@C_80_-*I*_h_ (M_2_ = Y_2_, DyY, and Dy_2_), *i.e.* the ratio between Y_2_ : DyY : Dy_2_ in fraction **F1**, is estimated using mass-spectrometry (Fig. 1, 1 : 2.4 : 3.6) and from the peak areas after recycling HPLC (Fig. 2a, 1 : 2.8 : 4.4). Two methods give consistent results, which deviate from the Y_2_ : DyY : Dy_2_ ratio of 1 : 2 : 1 expected for a purely statistical distribution given the Y : Dy ratio in the starting material is 1 : 1. A significant deviation indicates that Dy is more preferable for the formation of Ti-carbide clusterfullerenes than Y despite the slightly smaller ionic radius of the latter (0.90 Å for Y^3+^ and 0.91 Å for Dy^3+^ according to ref. 14). This finding also agrees with our previous observation that in the binary Dy–Ti and Y–Ti systems the yield of Dy_2_TiC@C_80_-*I*_h_ is higher than that of Y_2_TiC@C_80_-*I*_h_.^11*a*^

The central carbon in the endohedral carbide cluster bears a large negative charge similar to that in nitride clusterfullerenes, which results in a strong quasi-uniaxial ligand field and hence in a large magnetic anisotropy of the Dy ion(s) bonded to that carbon. Dy-based clusterfullerenes thus often behave as single molecule magnets.^4*a*,11*a*,13*b*,15^ Fig. 3a shows magnetization curves of DyYTiC@C_80_ measured between 1.8 and 8 K. The low-temperature curves show the butterfly-shaped magnetic hysteresis characteristic for single ion magnets exhibiting quantum tunneling of magnetization (QTM) near zero field.^16^ As intermolecular interactions are known to be one of the major perturbations causing the QTM in single-ion magnets, magnetization measurements were also performed for DyYTiC@C_80_ diluted in polystyrene (PS). Recently we showed that dilution in PS substantially reduces the QTM step in DySc_2_N@C_80_.^13*a*^ The decrease of the drop of the magnetization at zero-field caused by QTM is also observed in this work for PS-diluted DyYTiC@C_80_ (Fig. 3b). However, dilution in PS also leads to a strong diamagnetic background, which affects the shape of the magnetization curve. Besides, dilution increases the relaxation rate in a finite magnetic field.

Magnetic hysteresis is observed for DyYTiC@C_80_ up to 7 K, and is closed at higher temperatures. In agreement with these findings, the magnetic susceptibility measured during the temperature increase of a zero-field cooled sample (*χ*_ZFC_) and the magnetic susceptibility measured during cooling down the sample in a field of 0.2 T (*χ*_FC_) diverge below 8 K (Fig. 3a, inset). Interestingly, *χ*_ZFC_ shows not a sharp peak such as observed in DySc_2_N@C_80_ at 7.0 K,^13*a*^ but a broad peak with a plateau between 4.7 and 6.9 K. We thus determine the blocking temperature of magnetization of DyYTiC@C_80_ as *T*_B_ = 6.9 K. The overall magnetization behavior of DyYTiC@C_80_ and DySc_2_N@C_80_ is very similar. Both compounds exhibit butterfly hysteresis in the same temperature range with *T*_B_ values close to 7 K. However, a comparison of the magnetic hysteresis curves (Fig. 3b) shows that the hysteresis in DySc_2_N@C_80_ is broader, which points to a slower relaxation of the magnetization in the nitride clusterfullerene.

Relaxation times of the magnetization for DyYTiC@C_80_ were measured by first magnetizing the sample to saturation at 5 T, then sweeping the field fast to 0.2 T, and then recording a decay of the magnetization while the system was slowly restoring its equilibrium state. Decay curves were measured at several temperatures between 1.8 and 4 K and then fitted with a stretched exponential. Above 4 K the relaxation time of DyYTiC@C_80_ is shorter than 100 s, and the determination of the relaxation time by DC SQUID magnetometry is less reliable because of the finite field sweep rates and the time necessary for the stabilization of the magnetic field before recording the decay curve. A detailed discussion of the procedure can be found in ref. 13*a*. The low yield of the compound precluded an accumulation of the amounts necessary for measurements of shorter relaxation times by AC magnetometry.

The temperature dependence of the relaxation time of the magnetization in DyYTiC@C_80_ is plotted in Fig. 4. As QTM is quenched by a finite magnetic field of 0.2 T, a very slow decay of the magnetization with the relaxation time of 2.3 × 10^4^ s is observed at 1.8 K. An increase of the temperature accelerates the relaxation, and the temperature dependence takes a linear form in Arrhenius coordinates, which is usually associated with the Orbach relaxation mechanism:

(1) $$\tau^{-1}={\tau_{0}}^{-1}\;\text{exp}\left(-U^{\text{eff}}/T\right)$$

where *U*^eff^ is the effective barrier and *τ*_0_ is the attempt time. Fitting the relaxation times of DyYTiC@C_80_ with eqn (1) gives *U*^eff^ of 14.9 ± 0.3 K and *τ*_0_ of 6.5 ± 0.7 s. The relaxation of magnetization in DySc_2_N@C_80_ in this temperature range is also described by eqn (1) with *U*^eff^ = 23.6 ± 1 K and *τ*_0_ = 0.6 ± 0.2 s.^13*a*^ At 1.8 K its relaxation time is as long as 5.1 × 10^5^ s, which is 22 times longer than in DyYTiC@C_80_. But the higher effective barrier and shorter attempt time in DySc_2_N@C_80_ result in a faster temperature decay, and near 4 K the two EMFs exhibit similar relaxation times (Fig. 4). The nature of the low-temperature *U*^eff^ barriers of 15–25 K in EMF SMMs is not very clear. The crystal field splitting of Dy in clusterfullerenes is very strong and the energies of the spin excited states exceed hundreds of K.^6*c*,12,15*b*,17^ Besides, the *τ*_0_ values are many orders of magnitude longer than those usually found for the Orbach mechanism. We propose that the relaxation of magnetization in SMM-EMFs in this temperature range may follow the Raman mechanism with involvement of local vibrations of the endohedral cluster,^13*a*,15*a*,*b*^ which would also be described using eqn (1).^18^

It is quite remarkable that whereas single-ion magnets DyYTiC@C_80_ and DySc_2_N@C_80_ are not that different in their SMM properties, at least at low temperatures accessible for the current measurements, their dinuclear counterparts, Dy_2_TiC@C_80_ and Dy_2_ScN@C_80_, exhibit a stronger variation of the magnetic properties. The relatively narrow magnetic hysteresis in Dy_2_TiC@C_80_ is closing already near 3 K,^11*a*^ whereas the blocking temperature of magnetization in Dy_2_ScN@C_80_ is as high as 8 K.^12^ The central atoms in the cluster play a two-fold role in SMM properties. First, it is the source of the large single-ion magnetic anisotropy.^15*b*^ Second, it is a bridge between two Dy ions and hence plays a certain role in their exchange interactions. Replacement of a nitride ion by a carbide in the trimetal cluster can therefore affect both factors. Our study of DyYTiC@C_80_ and its comparison to DySc_2_N@C_80_ shows that the variation of the single-ion anisotropy appears to be of lesser importance for the low-temperature SMM behavior of the EMFs than the exchange coupling.

To conclude, in this work we synthesized the first carbide clusterfullerene with three different metals in the cluster, DyYTiC@C_80_-*I*_h_. The Ti-carbide template limits the possible range of compositions of the mixed-metal clusters, and EMFs with three different metals can be obtained relatively straightforwardly. This opens a way for combining metals with different functionalities within one molecule. We also showed that DyYTiC@C_80_-*I*_h_ is a single molecule magnet with QTM near zero field and a blocking temperature of magnetization at 7 K.

## Supplementary Material

† Electronic supplementary information (ESI) available: Experimental details, mass-spectra, and details of relaxation measurements. See DOI: 10.1039/c8cc04736g

SI

## Figures and Tables

**Fig. 1:**
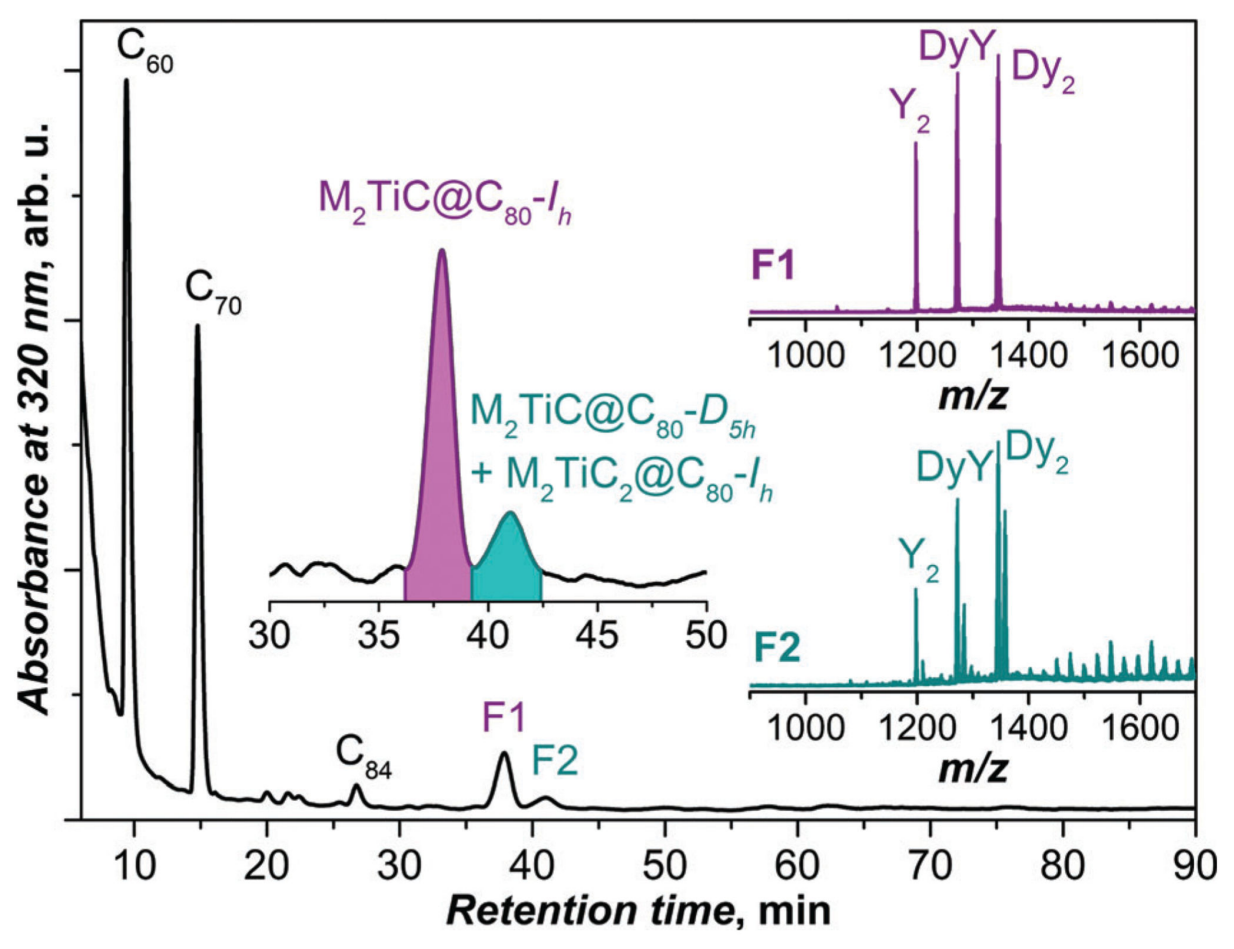
Representative HPLC chromatogram of the fullerene extract, obtained by the arc-discharge synthesis in the Dy–Y–Ti metal system and methane as a reactive gas (two analytical BuckyPrep columns, toluene as an eluent with a flow rate of 1.6 mL min^−1^ at 40 °C). The insets show an enhancement in the chromatogram in the range of the main EMF fractions **F1** and **F2** (shaded magenta and dark cyan, respectively) as well as their LDI mass-spectra (positive-ion mode).

**Fig. 2:**
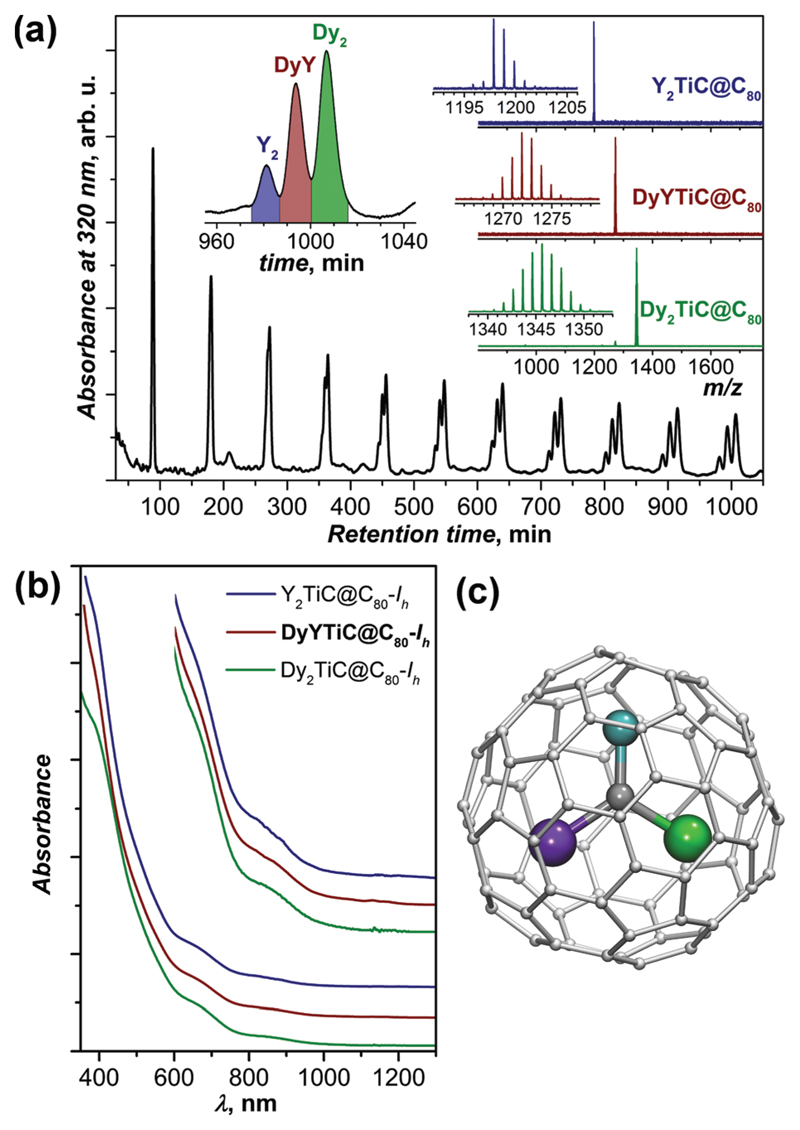
(a) Separation of the fraction **F1** (M_2_TiC@C_80_-*I*_h_) by recycling HPLC (semi-preparative BuckyPrep column, toluene as an eluent); the insets show the enhancement of the 11th cycle marking the composition of each component and mass-spectra of purified M_2_@TiC@C_80_-*I*_h_ fullerenes (M_2_ = Y_2_, DyY, and Dy_2_). (b) UV-vis-NIR absorption spectra of M_2_@TiC@C_80_-*I*_h_ fullerenes (M_2_ = Y_2_, DyY, and Dy_2_) in toluene (the inset shows the enhancement of the 600–1300 nm range). (c) Molecular structure of DyYTiC@C_80_-*I*_h_ (Dy is green, Y is violet, Ti is cyan, and carbons are grey).

**Fig. 3:**
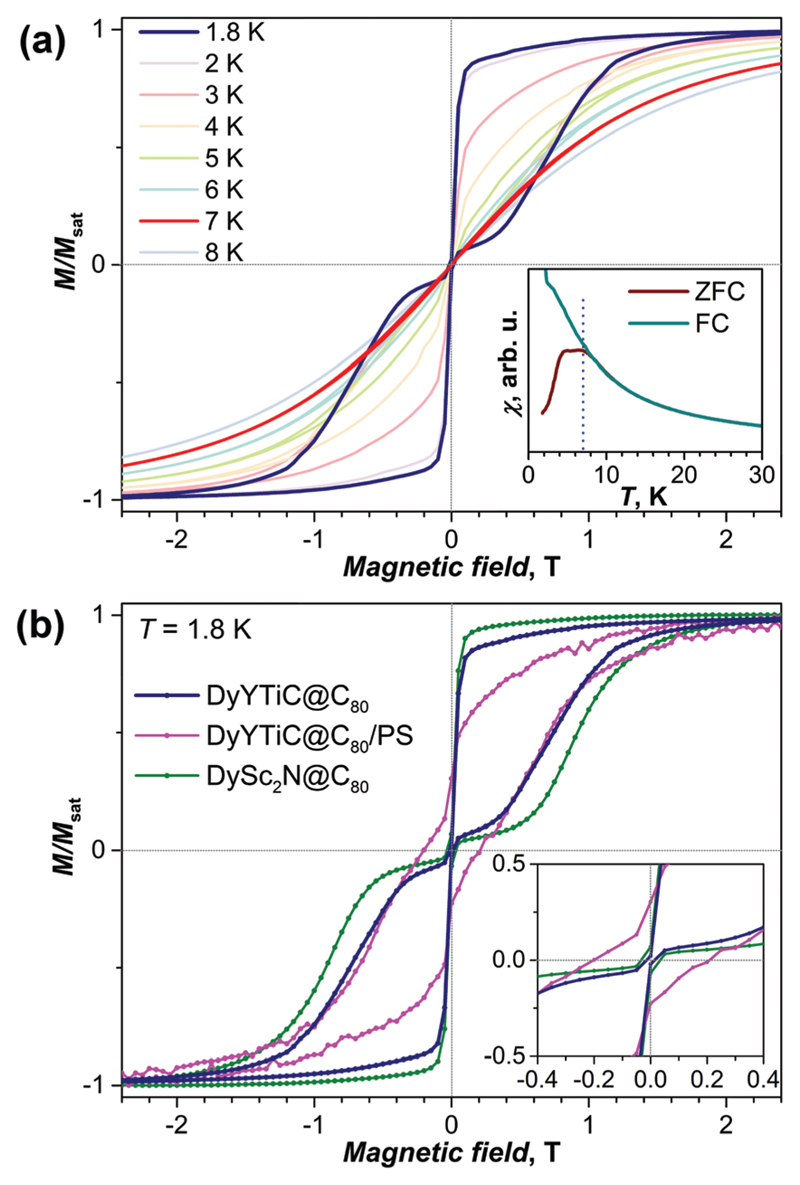
(a) Magnetization curves measured for DyYTiC@C_80_ at different temperatures; the inset shows the determination of the blocking temperature from the magnetic susceptibility (*χ*) measurements for zero-field cooled (ZFC) and in-field cooled (FC) samples (temperature sweep rate of 5 K min^−1^ in a field of 0.2 T). (b) Magnetic hysteresis at 1.8 K for non-diluted DyYTiC@C_80_, DyYTiC@C_80_ diluted in polystyrene (PS), and for the non-diluted DySc_2_N@C_80_; the inset shows enhancement of the region near zero field. Magnetic field sweep rate in all measurements is 2.9 mT s^−1^.

**Fig. 4:**
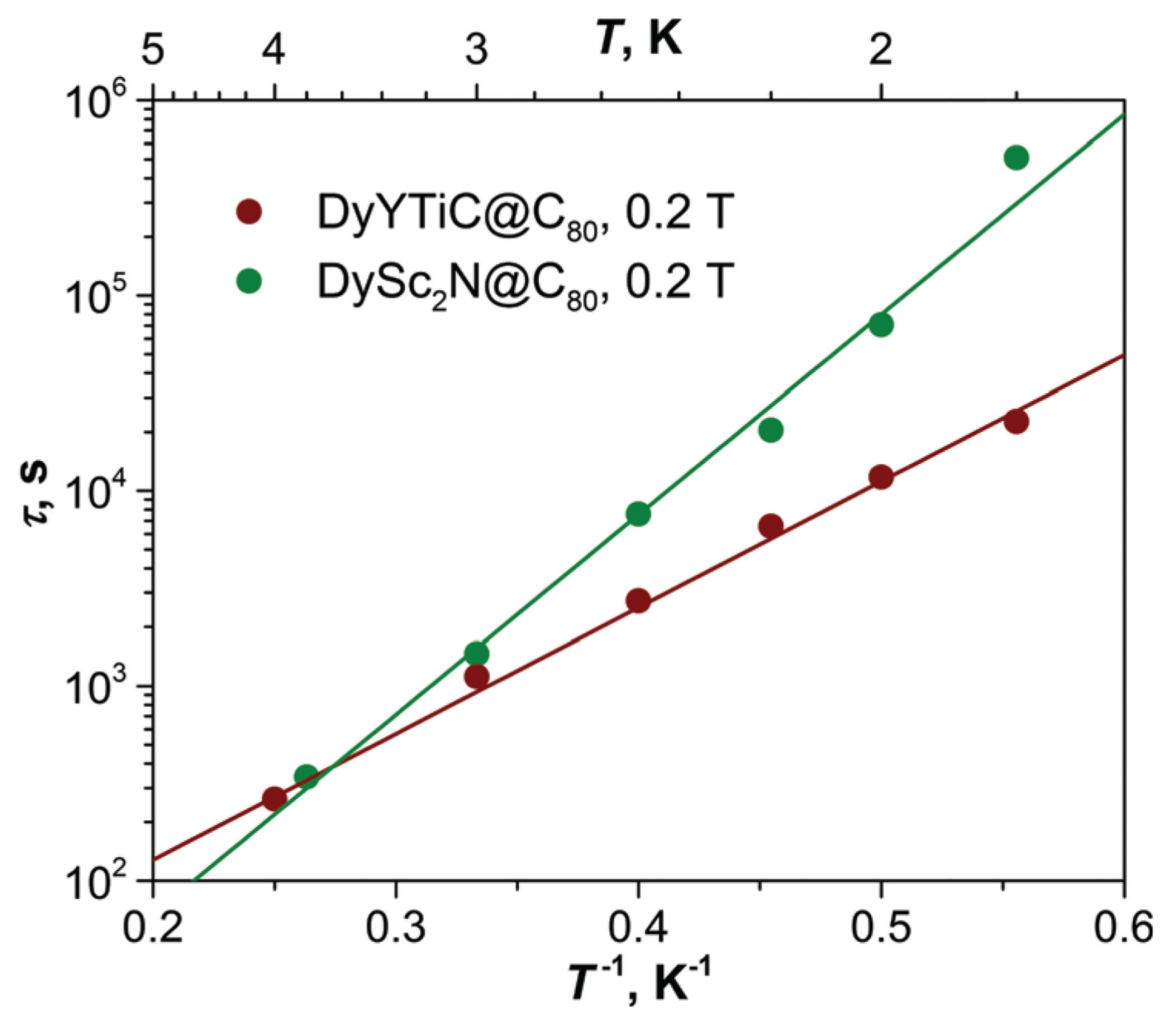
Relaxation times of magnetization of DyYTiC@C_80_ and DySc_2_N@C_80_ measured in the field of 0.2 T (dots) and their fits with eqn (1) (straight lines).
