# Supplementary material for: Carbide clusterfullerene DyYTiC@C80 featuring three different metals in the endohedral cluster and its single-ion magnetism†
Source: Chem Commun (Camb). Author manuscript; Available in PMC 2019 Nov 8. (PMC6839965; doi:10.1039/c8cc04736g)
Supplement: SI [file EMS84814-supplement-SI.pdf]

Electronic Supplementary Material (ESI) for ChemComm.
This journal is © The Royal Society of Chemistry 2018

Carbide clusterfullerene DyYTiC@C_{80} featuring three different metals in the endohedral cluster and its single-ion magnetism

Ariane Brandenburg, Denis S. Krylov, Alexander Beger, Anja U. B. Wolter, Bernd Büchner, and Alexey A. Popov

Supporting Information

Experimental details S2

Mass-spectra S3

Determination of the relaxation times S5

Magnetization decay curves S6

References S8

Experimental details

Synthesis. Graphite rods (6 x 10 mm) were core-drilled (diameter 4 mm), packed with a mixture of Dy, Y, Ti metal and graphite powder (molar ratio 0.5 : 0.5 : 1 : 12.5). The rods were then vaporized in a Krätschmer-Huffman-type fullerene generator with an arc current of 120 A applied in pulses of 30-60 s with changing of the polarity between each pulse (to ensure evaporation of both rods). The atmosphere in the reactor chamber contained 237 mbar helium and 13 mbar methane. Methane is used here to suppress formation of empty fullerenes and of monometallofullerenes and hence to increase the relative yield of carbide clusterfullerenes and to facilitate their HPLC separation. The influence of methane on the relative yield of different fullerenes was studied in refs. [1]

The collected soot was Soxhlet-extracted with carbon disulfide for 20 h. The extract was dried by CS_2 distillation. The solid residue was dissolved in toluene and filtered. The desired $M_2TiC@C_{80}$-I_h compound were isolated from empty fullerenes and other endohedral fullerenes by two-stage HPLC process. In the first stage, the toluene solution of the extract was separated by HPLC monitored using a UV detector at 320 nm and a linear combination of two analytical 4.6 mm × 250 mm Buckyprep columns (Nacalai Tesque, Japan) with toluene as the mobile phase (Fig. 1). In the second stage, fraction **F1** was subjected to recycling HPLC with a 10 × 250 mm Buckyprep column (Nacalai Tesque, Japan), resulting in the isolation of $Y_2TiC@C_{80}$-I_h, $DyYTiC@C_{80}$-I_h, and $Dy_2TiC@C_{80}$-I_h (Fig. 2).

To accumulate ca 1 mg of the mixture of $Y_2TiC@C_{80}$-I_h, $DyYTiC@C_{80}$-I_h, and $Dy_2TiC@C_{80}$-I_h (i.e. fraction **F1**), 20 arc-discharge syntheses were needed. In each synthesis, two graphite rods packed with metal mixture are evaporated. Each drilled graphite rod weighed 3±0.2 g and was filled with 1±0.1 g of the mixture containing graphite and metal powders (0.46 g graphite powder, 0.25 g Dy, 0.14 g Y, and 0.15 g Ti). The mass of the pure $DyYTiC@C_{80}$-I_h after recycling HPLC separation of the fraction **F1** was ca 0.2 mg.

LDI mass spectra were recorded on a Bruker autoflex mass-spectrometer.

UV-vis-NIR absorption spectra were measured in toluene solution at room temperature with Shimadzu 3100 spectrophotometer.

DC magnetization measurements were performed using a Quantum Design VSM MPMS3 magnetometer.

Mass-spectra of isolated $M_2TiC@C_{80}$ compounds

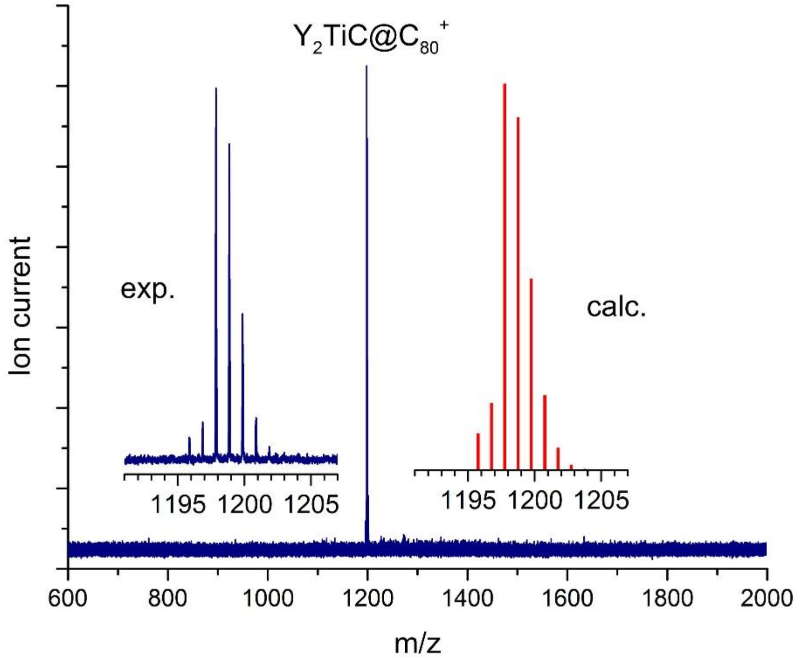

Fig. S1. Mass-spectrum of isolated $Y_2TiC@C_{80}$. The insets show experimental and calculated isotopic distribution

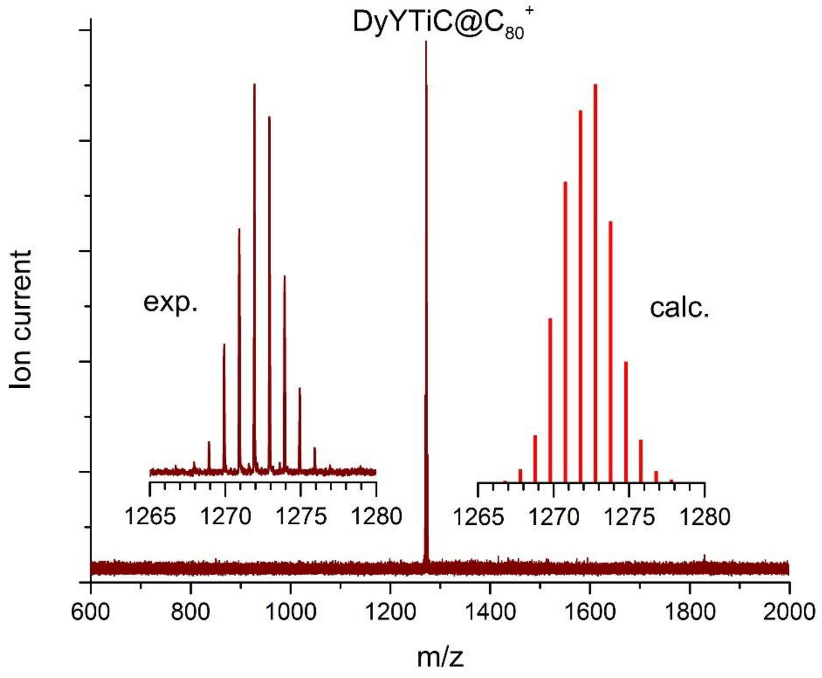

Fig. S2. Mass-spectrum of isolated $DyYTiC@C_{80}$. The insets show experimental and calculated isotopic distribution

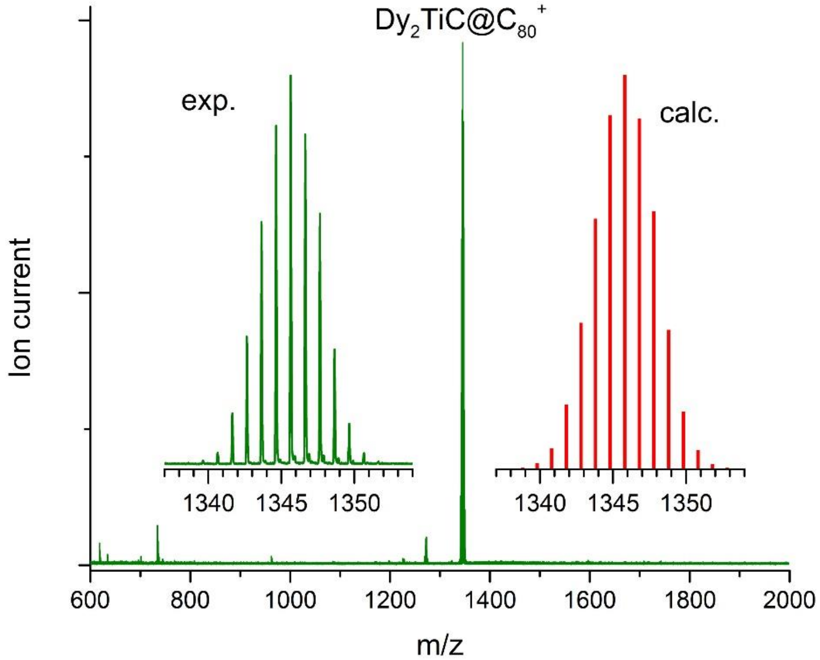

Fig. S3. Mass-spectrum of isolated $Dy_2TiC@C_{80}$. The insets show experimental and calculated isotopic distribution.

Determination of relaxation times

The DC measurement of the magnetization decay curve is relatively simple and doesn't require large sample amounts (5-10 % compared to AC). However, difficulties arise during fitting of the experimental decay curves as single-molecule magnets tend to exhibit a time-dependent decay rate. One of the reasons is arising from the evolution of internal dipolar fields in the sample during relaxation. Consequently a single exponential function often fails to describe the system's behavior. In a general case the decay curve consists of an infinite number of exponentials, and characteristic value for the relaxation time distributions has to be derived. It becomes possible with a stretched exponential:

$$f(t) = M_{eq} + (M_0 - M_{eq})\, exp\left[-\left(\frac{t}{\tau_1}\right)^{\beta}\right] \quad \text{(S.1)}$$

Where M_{eq} and M_0 are the equilibrium and initial magnetizations, respectively, τ_1 is a characteristic "average" relaxation time and β is an additional parameter that corresponds to the time-dependent decay rate $\tau^{-1} \sim t^{\beta-1}$ with $\beta = (0; 1)$. In the extreme case of $\beta = 1$ one obtains a single exponential.

When relaxation time is considerably longer than the reasonable measurement time, it becomes problematic to determine the equilibrium magnetisation in Eq. S.1 for such incomplete curves, and the results of the fit strongly depend on correct determination of this value. In order to increase the reliability of the fit, an additional "thermal" magnetisation curve has been recorded for such cases. With the magnetic field kept at the required value, one warms the sample above the blocking temperature and then cools it down to the initial temperature, where the measurement starts. The magnetisation is rising towards the same M_{eq} as for the decay curve. A combined fitting of two curves yields more accurate results as described in the Ref. [2].

Table S1. Relaxation times and β-parameters from stretched exponential fitting of magnetization decays curves measured for DyYTiC@C_{80}-*I*$_h$ in a field of 0.2 T.

T, K	*τ*, s	st. dev. *τ*	β	st. dev. β
1.8	22731.5	124.5	0.438	0.001
2.0	11770.0	129.4	0.422	0.003
2.2	6578.9	40.6	0.363	0.003
2.5	2721.6	26.9	0.344	0.002
3.0	1118.6	6.3	0.498	0.002
4.0	262.6	0.5	0.705	0.001

Magnetization decay curves

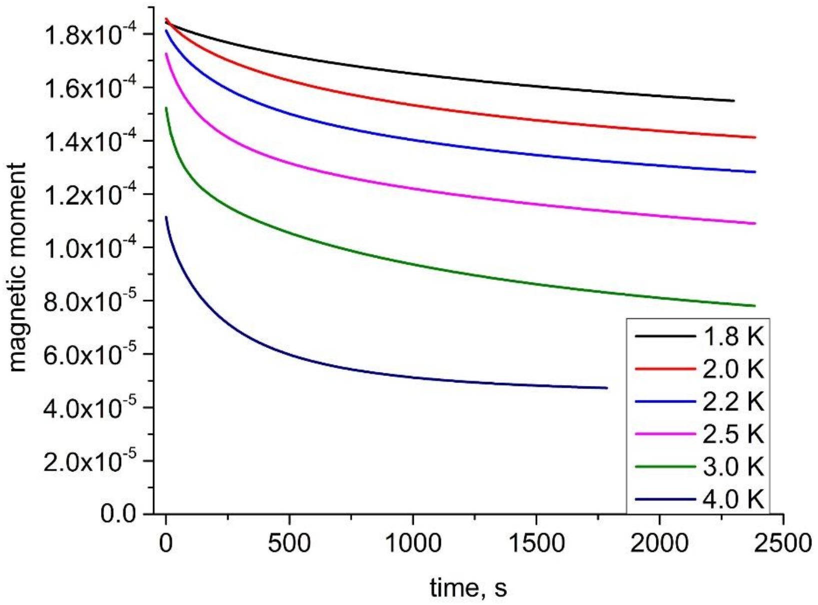

Figure S4. Magnetization decay curves measured for DyYTiC@C_{80}-I_h at different temperatures in a field of 0.2 T

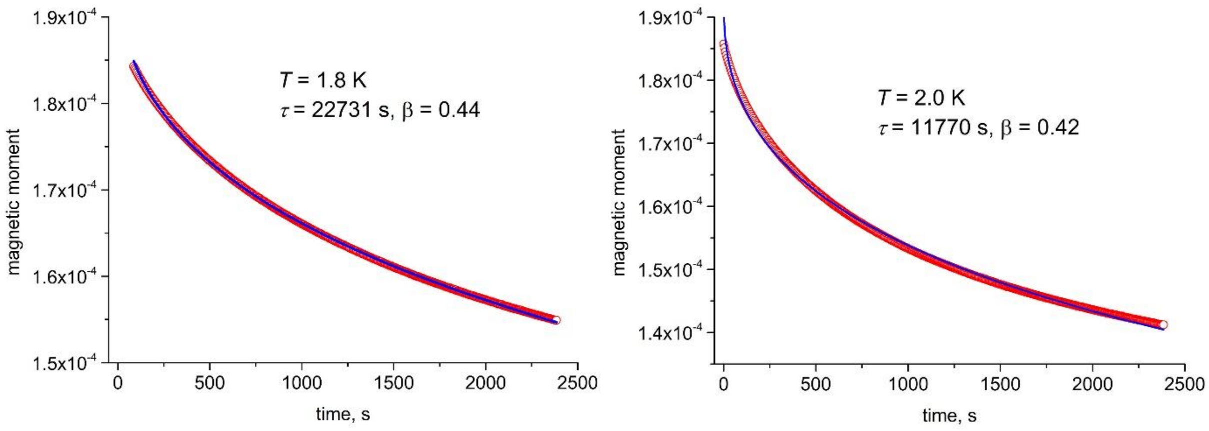

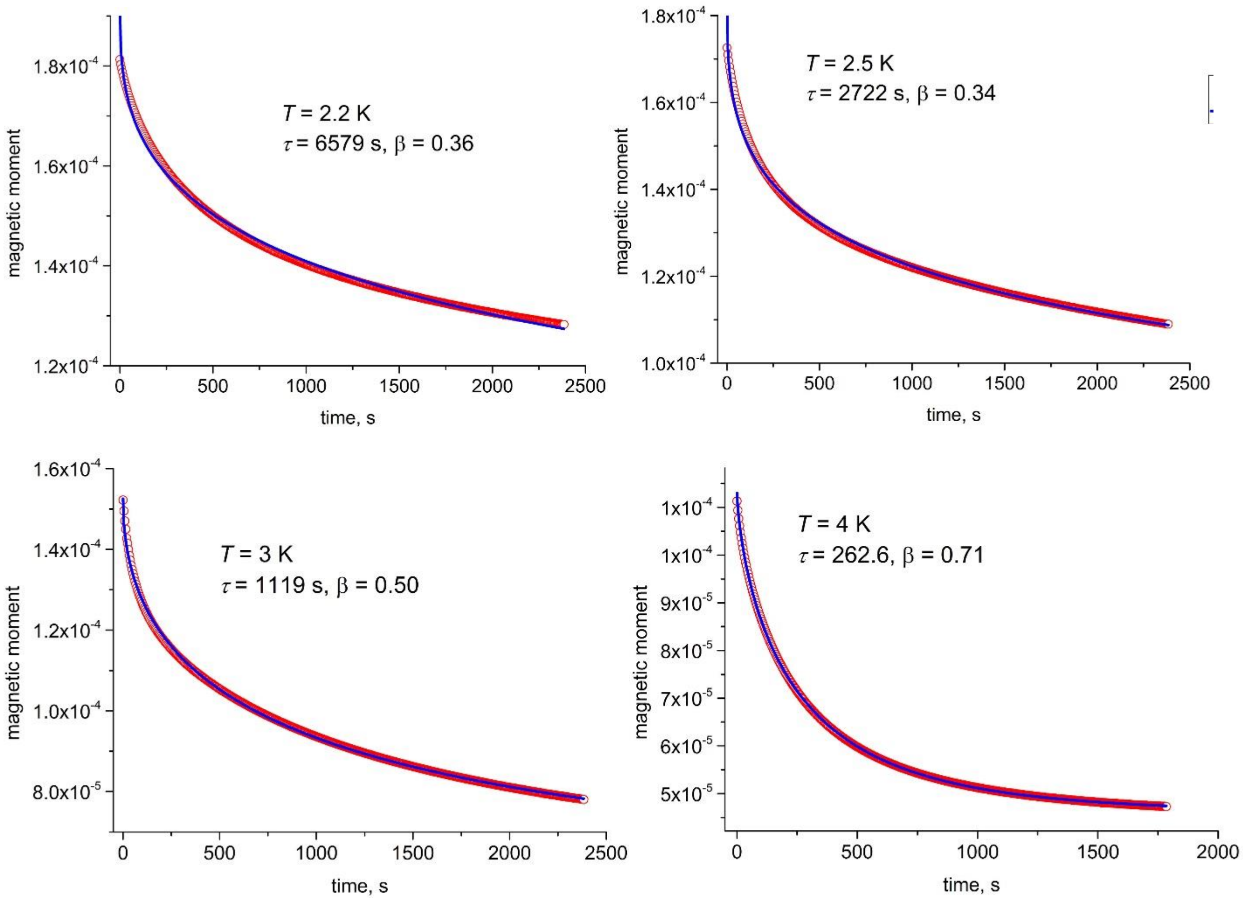

Figure S5. Fitting of experimental decay curves with stretched exponentials for DyYTiC@C_{80}-I_h

References

1. a) K. Junghans, M. Rosenkranz and A. A. Popov, *Chem. Commun.*, 2016, **52**, 6561; b) K. Junghans, K. B. Ghiassi, N. A. Samoylova, Q. Deng, M. Rosenkranz, M. M. Olmstead, A. L. Balch and A. A. Popov, *Chem.-Eur. J.*, 2016, **22**, 13098; c) Q. Deng, K. Junghans and A. A. Popov, *Theor. Chem. Acc.*, 2015, **134**, 10; d) K. Junghans, C. Schlesier, A. Kostanyan, N. A. Samoylova, Q. Deng, M. Rosenkranz, S. Schiemenz, R. Westerström, T. Greber, B. Büchner and A. A. Popov, *Angew. Chem. Int. Ed.*, 2015, **54**, 13411; e) A. L. Svitova, K. Ghiassi, C. Schlesier, K. Junghans, Y. Zhang, M. Olmstead, A. Balch, L. Dunsch and A. A. Popov, *Nat. Commun.*, 2014, **5**, 3568; f) C.-H. Chen, D. S. Krylov, S. M. Avdoshenko, F. Liu, L. Spree, R. Yadav, A. Alvertis, L. Hozoi, K. Nenkov, A. Kostanyan, T. Greber, A. U. B. Wolter and A. A. Popov, *Chem. Sci.*, 2017, **8**, 6451.
2. D. Krylov, F. Liu, A. Brandenburg, L. Spree, V. Bon, S. Kaskel, A. Wolter, B. Buchner, S. Avdoshenko and A. A. Popov, *Phys. Chem. Chem. Phys.*, 2018, **20**, 11656.
